# CR-GCN: Channel-Relationships-Based Graph Convolutional Network for EEG Emotion Recognition

**DOI:** 10.3390/brainsci12080987

**Published:** 2022-07-26

**Authors:** Jingjing Jia, Bofeng Zhang, Hehe Lv, Zhikang Xu, Shengxiang Hu, Haiyan Li

**Affiliations:** 1 School of Computer Engineering and Science, Shanghai University, Shanghai 200444, China; jiajingjing@shu.edu.cn (J.J.); hhlv@shu.edu.cn (H.L.); xuzhikangnba@shu.edu.cn (Z.X.); shengxianghu@shu.edu.cn (S.H.); 2 School of Computer and Communication Engineering, Shanghai Polytechnic University, Shanghai 201209, China; 3 School of Computer Science and Technology, Kashi University, Kashi 844008, China; lihaiyan_2016@sjtu.edu.cn

**Keywords:** electroencephalography, emotion recognition, adjacency matrix, CR-GCN

## Abstract

Electroencephalography (EEG) is recorded by electrodes from different areas of the brain and is commonly used to measure neuronal activity. EEG-based methods have been widely used for emotion recognition recently. However, most current methods for EEG-based emotion recognition do not fully exploit the relationship of EEG channels, which affects the precision of emotion recognition. To address the issue, in this paper, we propose a novel method for EEG-based emotion recognition called CR-GCN: Channel-Relationships-based Graph Convolutional Network. Specifically, topological structure of EEG channels is distance-based and tends to capture local relationships, and brain functional connectivity tends to capture global relationships among EEG channels. Therefore, in this paper, we construct EEG channel relationships using an adjacency matrix in graph convolutional network where the adjacency matrix captures both local and global relationships among different EEG channels. Extensive experiments demonstrate that CR-GCN method significantly outperforms the state-of-the-art methods. In subject-dependent experiments, the average classification accuracies of 94.69% and 93.95% are achieved for valence and arousal. In subject-independent experiments, the average classification accuracies of 94.78% and 93.46% are obtained for valence and arousal.

## 1. Introduction

Emotion recognition is a significant research direction in affective computing, which is the main technology to achieve high-level human–computer interaction. The purpose of emotion recognition is to allow machines to perceive human emotional states, so as to enhance the humanization level of machines [1,2]. In addition, this type of study may also be used to understand humans [3]. Traditional emotion recognition methods mostly use easily accessible facial expression images [4], body gestures, and speech signals [5,6]. However, the validity and reliability of nonphysiological signals are often difficult to guarantee in practical applications [7]. Physiological signals, such as EEG, electrooculogram, and electromyography, are not easily controlled by subjective consciousness and have been shown to reveal important information about human emotional states [8,9]. In recent years, EEG-based emotion recognition has attracted more and more attention in both research [10,11] and applications [12].

At present, the research on emotion model mainly includes two categories: discrete method and dimension method [13]. The discrete method classifies emotions into discrete status, as Ekman et al. [14] classified emotions into joy, sadness, surprise, fear, anger, and disgust. The dimension method describes emotions as two dimensions (valence and arousal) or three dimensions (valence, arousal, and dominance) [15].

Many researchers have used deep learning for EEG-based emotion recognition. Tripathi et al. [16] used a convolutional neural network (CNN) to extract features of different EEG channels to realize emotion recognition. However, in fact, the distribution of EEG channels is not gridlike but is irregularly connected. In recent decades, graph convolutional network (GCN) has been shown to effectively use adjacency matrices to capture interchannel relationships and extract graph domain features to realize emotion recognition. Zhong et al. [17] and Yin et al. [18] employed a distance-based method to exploit the relationship among EEG channels using GCN. However, these methods only capture interchannel relationships in a single way and do not fully exploit the relationship of EEG channels, which affects the precision of emotion recognition. Neuroscience studies have shown that emotional patterns are related to functional connectivity of brain regions [19,20].

To address the problem, we propose a new channel-relationships-based graph convolutional network (CR-GCN) method by exploiting the relationships among EEG channels. Specifically, 3 s of baseline EEG data of the subjects collected before watching the video is first used to eliminate the noise that the brain produces spontaneously. Then, a time window of 6 s is used to partition the data. From each segment, power spectral density (PSD) is extracted and normalized to construct feature cube. Second, an adjacency matrix is constructed using both the topological structure and functional connectivity of EEG channels simultaneously. Third, the feature cube of each segment and adjacency matrix are used as the input of GCN model, and softmax layer outputs are used to predict classification results. The major contributions of our paper are as follows.

- A novel emotion recognition method by exploiting multiple relationships among EEG channels is proposed. The topological structure of EEG channels represents local relationships, and the brain functional connectivity represents global relationships. Our method combines both relationships, which captures both local and global relationships among EEG channels, and can more accurately reflect the interaction between EEG signals.
- A fusion method for relationships among EEG channels is proposed. Graph is used to represent the topological relationship and functional connectivity relationship of EEG channels. EEG channel relationships are constructed by an adjacency matrix in GCN. The adjacency matrix is constructed from the corresponding adjacency matrices represented by two graphs.
- Experimental results demonstrate that the CR-GCN method achieves better classification results than the state-of-the-art methods.

## 2. Related Work

### 2.1. Features of EEG Signals

Musha et al. [21] were the first to use EEG signals to realize emotion recognition and extracted features from ten channels. In recent decades, many methods have been used to extract EEG features [22,23]. The features extracted from EEG signals mainly include three categories: time domain, frequency domain, and time–frequency domain. Time domain features include Hjorth feature [24], high-order crossing feature [25], and fractal dimension feature [26]. Frequency domain features include differential asymmetry (DASM) [26], differential entropy (DE) [27,28], PSD [28], approximate entropy [29], sample entropy [30], and rational asymmetry (RASM) [31]. Commonly used time–frequency domain features are short-time Fourier transform [32] and wavelet transform [33].

Inspired by several studies [34,35,36], PSD is used to extract EEG features in this paper. According to the relevant biological study [2], the higher frequency bands (such as theta, alpha, beta, and gamma band) are more associated with emotional activities, while the lower frequency bands (such as delta band) are less associated with emotional activities. Therefore, this paper uses theta, alpha, beta, and gamma band to extract PSD.

### 2.2. Graph Convolutional Network

Traditional CNN is limited in handling irregular and non-Euclidean domain data. Compared with it, GCN [37] can handle irregular and non-Euclidean domain data, so it has more advantages in processing discrete spatial domain signals [38]. More importantly, GCN has been shown to effectively use adjacency matrices to capture interchannel relationships and extract graph domain features to realize emotion recognition [17,18,34,35,39]. Many researchers have used GCN for EEG emotion recognition. Song et al. [34] designed a dynamical graph convolutional neural network (DGCNN) to exploit the relationship between irregular EEG channels but dynamically updating the adjacency matrix resulted in more model parameters and a long calculation period. Zheng et al. [35] extracted six types of features from five frequency bands and input these features into a hierarchical graph convolutional network (HGCN), but HGCN only considered the horizontal and vertical relationship of the overall EEG channels and did not specifically exploit the relationship between individual channels. Zhong et al. [17] introduced a regularized graph neural network (RGNN) by considering the biological topology and brain asymmetry to exploit the local and global relationships of different channels to construct an adjacency matrix to realize emotion recognition, but they did not take into account the functional connectivity of brain channels. Yin et al. [18] designed a novel emotion recognition method based on a deep learning model (ERDL) by extracting differential entropy from EEG data to construct feature cubes and used GCN and long short-term memory (LSTM) to realize emotion recognition, but they only used the distance to design the interchannel relationship. Inspired by DGCNN [34], Jin et al. [39] applied GCN with learnable EEG electrode relationships in a goal-driven manner for emotion recognition, but it still needed a great amount of calculation to determine the adjacency matrix every time.

Although the above methods have applied GCN to realize emotion recognition and used adjacency matrices to capture interchannel relationships, they have not fully exploited the interchannel relationships. Therefore, this paper attempts to design a method that can more accurately reflect the interaction between EEG signals to improve the accuracy of emotion recognition.

## 3. CR-GCN Method

**Figure 1:**
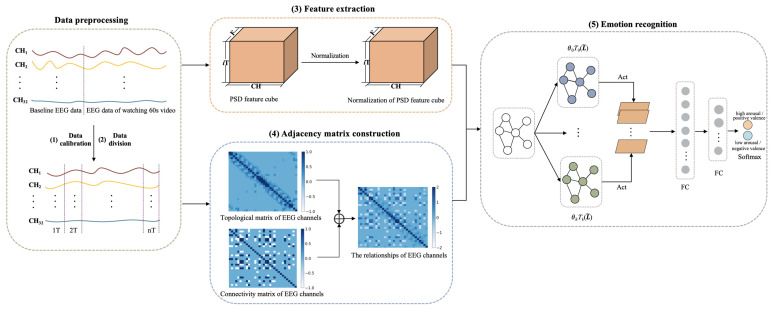
Framework of CR-GCN. Act denotes ReLU activation. FC denotes full connection.

The framework of CR-GCN is shown in Figure 1. The CR-GCN method includes five parts.

(1) Data calibration. First, 3 s of baseline EEG data of the subjects collected before watching the video is averaged and then replicated 20 times to form the 60 s data. Then, the corresponding baseline data is subtracted from the EEG data of watching 60 s video. With this, EEG signals have a high probability of removing noise signals that are not related to emotions [18,40].

(2) Data division. After (1), the data is partitioned into ((60−T)/S+1) segments for each video where *T* and *S* are respectively set to 6 and 3 in the following experiments.

(3) Feature extraction. PSD is adopted to extract EEG features in this work. We introduce a method for normalization of EEG features of subjects for better emotion recognition.

(4) Adjacency matrix construction. The topological matrix of EEG channels is the distance-based method and tends to study local relationships among EEG channels. The connectivity matrix of EEG channels is based on the functional connectivity method and tends to study global relationships among EEG channels. Therefore, in order to describe the relationship among EEG channels more accurately, we propose a method that combines distance and functional connectivity among channels to construct the adjacency matrix, which captures both local and global relationships among EEG channels.

(5) Emotion recognition. This paper adopts the GCN for emotion recognition, in which the normalization of PSD feature cube is used as the node representation, and the adjacency matrix is served as the node relationships. The softmax layer outputs are used to predict emotion classification results.

### 3.1. Feature Extraction

PSD is used to calculate the signal power in different frequency bands according to Fourier transform, which is widely used in signal processing. Suppose we have a power signal *f*(*t*); in order to be able to perform Fourier transform on it, we intercept a section with a time length of 2*T* and perform Fourier transform on the signal within this time period. It is defined in Equation (1):

(1) $$F_{T}\left(\omega \right)=\int_{-\infty }^{\infty }f_{T}\left(t\right)e^{j2\pi \omega t}dt=\int_{-T}^{T}f\left(t\right)e^{j2\pi \omega t}dt$$

According to the Parseval formula, we can get Equation (2)

(2) $$\int_{-T}^{T}f{\left(t\right)}^{2}dt=\int_{-\infty }^{\infty }{\left|X_{T}\left(\omega \right)\right|}^{2}d\omega$$

where $X_{T}\left(w\right)$ =$F\left[f(t)\right]$, F[] denotes the Fourier transform.

When the time *T* tends to infinity, it can be known that $f_{T}$(*t*) can be approximately equivalent to *f*(*t*), and $F_{T}$(*w*) can also be equivalent to *F*(*w*). So, we take the time of the formula toward infinity and divide by 2π*T*. The formula for calculating the PSD can be obtained as follows:

(3) $$P\left(w\right)=\frac{1}{2\pi T}\int_{-\infty }^{\infty }\lim_{T\to \infty }{\left|X_{T}\left(\omega \right)\right|}^{2}d\omega =\lim_{T\to \infty }\frac{{\left|X_{T}\left(\omega \right)\right|}^{2}}{2\pi T}$$

(4) $$PSD=\lim_{T\to \infty }\frac{{\left|X_{T}\left(w\right)\right|}^{2}}{2\pi T}$$

According to the characteristics of EEG signals, in this paper, we extract PSD from four bands; then, we calculate the average value of PSD in different frequency bands and then normalize PSD. The standard deviation and the normalization formula are respectively given in Equations (5) and (6):

(5) $$\sigma =\sqrt{\frac{1}{N}\sum_{i=1}^{N}{\left(x_{i}-\mu \right)}^{2}}$$

(6) $$z=\frac{x-\mu }{\sigma }$$

where σ is the standard deviation of PSD of frequency band, *N* is the number of samples, $x_{i}$ is the value of PSD of the frequency band of each sample, μ is the mean value of PSD of the frequency band, *z* is the normalized PSD, and *x* is the value of PSD of the frequency band.

### 3.2. Design of Channel Relationships Based GCN

#### 3.2.1. Graph Representation

Inspired by DGCNN [34] and RGNN [17], in this paper, each EEG channel is represented as a node in a graph. The graph can be defined as *G* = {*V*, ε, *A*}, where *V* is a set of nodes, ε is a set of edges between nodes in *V*, and $A\in R^{N\times N}$ is the adjacency matrix. **A** represents the relationship of EEG channels and *N* represents the number of EEG channels. The value $A_{ij}$ is learnable and represents the relationship between node *i* and *j*.

**Figure 2:**
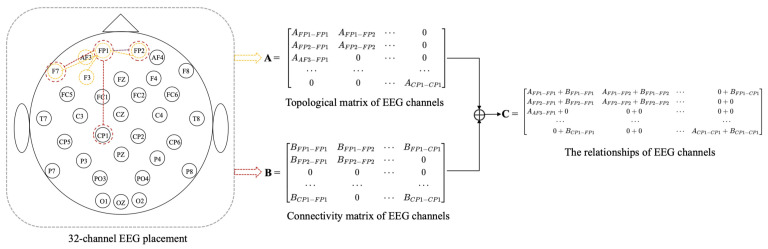
Two-dimensional placement for 32-channel EEG and adjacency matrix construction.

There are three methods to calculate the value of $A_{ij}$, such as functional connectivity [41], distance-based [17,18], and neural network. EEG can be described as the result of randomly distributed dipoles [42], and the dipole-driven nature of the EEG results in electrodes that are associated with distant electrodes. Since the adjacency matrix constructed by distance-based method [17,18] only considers the relationship between electrodes within a short distance and ignores there are also correlations between distant electrodes. Therefore, we propose a method that combines distance-based method and functional connectivity among channels to construct the adjacency matrix, in which it captures both local and global relationships of EEG channels. To better describe the position and adjacency matrix correspondence, the two-dimensional locations of EEG electrodes and adjacency matrix construction are shown in Figure 2. We construct adjacent matrix as a symmetric matrix with at most (*N* + 1)*N*/2 parameters instead of $N^{2}$ to reduce overfitting.

(1) Topological matrix of EEG channels. The construction of the adjacency matrix **A** is the distance-based method. We construct local relationships using distance-based method among EEG channels in the adjacency matrix **A**. In this paper, **A** is constructed by calculating the Euclidean distance between EEG channels in 3D space, and the 3D coordinates of EEG channels can be obtained from the recorded EEG data. Salvador et al. [43] found that the strength of connectivity between brain regions was generally related to distance by an inverse square law. Zhong et al. [17] and Yin et al. [18] have used distance-based method to construct adjacency matrix. Therefore, the formula for constructing $A_{ij}$ is as follows:

(7) $$A_{ij}=\min\left(1,\frac{\delta }{d_{ij}^{2}}\right)$$

where $d_{ij}$, *i, j* = 1, 2, …, *n*, represents Euclidean distance between nodes *i* and *j*, and δ represents calibration constant. According to the relevant study [44], retaining 20% of connections can improve the efficiency of model. Thus, δ is set to be the same as [17], so that about 20% of connections in **A** will be retained. We also treat the connections with values > 0.1 as nonignorable connections.

(2) Connectivity matrix of EEG channels. The construction of the adjacency matrix **B** is based on the functional connectivity method. We construct global relationships using the functional connectivity method among EEG channels in the adjacency matrix **B**. In this paper, adjacency matrix **B** of graph *G* is constructed based on the similarity between nodes. The similarity between nodes is calculated as follows:

(8) $$p_{xy}=\frac{\mathrm{cov}(x,y)}{\delta x\delta y}=\frac{E\left[\left(x-x_{\mu }\right)\left(y-y_{\mu }\right)\right]}{\delta x\delta y}$$

where functions cov and *E* denote covariance and expectation, respectively, *x* and *y* denote the values of the corresponding node, $x_{\mu }$ and $y_{\mu }$ denote the mean value of eigenvectors, and δx and δy denote the variance.

The formula for constructing $B_{ij}$ is defined as

(9) $$B_{ij}=\left\{\begin{array}{cc} p_{ij}, & \;\mathrm{if}\;|p_{ij}|>\lambda \\ 0, & \;\mathrm{otherwise}\; \end{array}\right.$$

where $B_{ij}$, *i, j* = 1, 2, …, *n*, represents the correlation coefficient between nodes *i* and *j*, and λ is set to the correlation coefficient threshold. If the absolute value of the correlation between nodes > λ, the connection will be retained. After repeated experiments, with the increase of λ, the accuracy of the model is higher. When λ is greater than 0.98, the accuracy of the model decreases significantly. λ is set to 0.98 in the following experiments.

Our method based on the combination of distance and functional connectivity among EEG channels is proposed to construct adjacency matrix **C** by adding Equations (7) and (9) to perform matrix fusion. The adjacency matrix **C** is based on the fusion of local and global connectivity.

#### 3.2.2. Spectral Graph Filtering

Spectral graph filtering is called graph convolution, which is a common signal processing method for graph data operation. Let **L** represent the standard Laplacian matrix of graph *G*. It is defined as

(10) $$L=E-D^{-\frac{1}{2}}CD^{-\frac{1}{2}},C\;\mathrm{and}\;D\in R^{N\times N}$$

where **D** is a diagonal matrix, $D_{ii}=\sum_{j}C_{ij}$, and **E** is an identity matrix.

The decomposition of **L** is as follows:

(11) $$L=U\Lambda U^{T}$$

where $U=\left(u_{1},u_{2},\ldots ,u_{n}\right)\in R^{N\times N}$ is the eigenvector matrix of Laplacian matrix **L**. $\Lambda =\mathrm{diag}\left(\lambda_{0},\lambda_{1},\ldots \lambda_{n}\right)\in R^{N\times N}$ is the eigenvalue diagonal matrix. $U_{i}\in R^{N},$i=1,2,…,n, is the eigenvector of **L**, and $\lambda_{i}$ is the eigenvalue of **L**.

The convolution of signals **x** and **y** on graph *G is as follows

(12) $$x*Gy=U\left(\left(U^{T}x\right)⊙\left(U^{T}y\right)\right)$$

Using *g*(·), **x** can be filtered as follows:

(13) $$y=g\left(L\right)x=Ug\left(\Lambda \right)U^{T}x$$

where g(Λ) is as follows:

(14) $$g\left(\Lambda \right)=\left[\begin{array}{ccc} g\left(\lambda_{1}\right) & \cdots & 0\\ \vdots & \ddots & \vdots \\ 0 & \cdots & g\left(\lambda_{N}\right) \end{array}\right]$$

Using *K*-order Chebyshev polynomials, we can get g(Λ), which is

(15) $$g\left(\Lambda \right)=\sum_{k=0}^{K-1}\theta_{k}T_{k}\left(\tilde{\Lambda }\right)$$

where $\theta_{k}$ is the coefficient of the Chebyshev polynomial and $T_{k}$(·) is the calculation method of the Chebyshev polynomial. The formula is as follows:

(16) $$\begin{array}{c} T_{0}\left(x\right)=1\\ T_{1}\left(x\right)=x\\ T_{k}\left(x\right)=2xT_{k-1}\left(x\right)-T_{k-2}\left(x\right),k\geq 2 \end{array}$$

Using (15), (13) can be transformed as

(17) $$\begin{array}{cc} y & =Ug\left(\Lambda \right)U^{T}x\\ \; & =\sum_{k=0}^{K-1}U\left[\begin{array}{ccc} \theta_{k}T_{k}\left(\lambda_{1}\right) & \cdots & 0\\ \vdots & \ddots & \vdots \\ 0 & \cdots & \theta_{k}T_{k}\left(\lambda_{N}\right) \end{array}\right]U^{T}x\\ \; & \approx \sum_{k=0}^{K-1}\theta_{k}T_{k}\left(\tilde{L}\right)x \end{array}$$

where $\tilde{L}=\frac{L}{\lambda_{MAX}}-E$. ChebNet [45] does not require feature decomposition of the Laplacian matrix, and the convolution kernel has only *K*+1 learnable parameters. The complexity of the parameters is significantly reduced, and the operation speed can be greatly improved.

### 3.3. Algorithm of CR-GCN

The loss function of CR-GCN is as follows

(18) $$loss=cross\_entropy(l,l^{p})+\alpha ||w||$$

where **l** represents the actual label vector, $l^{p}$ represents the predicted one, **w** represents the parameters of the model, α is the regularization coefficient, and α||w|| regularization term is designed to reduce overfitting. The algorithm of CR-GCN is shown in Algorithm 1.
**Algorithm 1:** The algorithm of CR-GCN    **Input**: EEG features, class labels, the number of Chebyshev polynomial *K*, the number of iterations MAX, learning rate η, stop iteration threshold *e*;    **Ouput**: The desired parameters of CR-GCN;
**1** **for**i=1,2,…,n**do**(**2** Calculate the adjacency matrix **C** according to Equations (7) and (9);**3** Calculate the standard Laplacian matrix **L** based on Equation (10);**4** Calculate the Chebyshev polynomial items (k=0,1,⋯,K−1) according to Equation (17);**5** Calculate $\sum_{k=0}^{K-1}\theta_{k}T_{k}\left(\tilde{L}\right)x$;**6** **end****7** epoch = 0;**8** **while**Loss > *e* || epoch < MAX do**9**       Calculate convolution results;**10**       Calculate the results of FC layer as shown in Figure 1;**11**       Calculate the loss according to Equation (18);**12**       Update graph convolution parameters;**13**       epoch = epoch + 1;**14** **end while**


## 4. Experiments

### 4.1. Dataset

A database for emotion analysis using physiological signals (DEAP) [46] is published for study of human emotional states. In this dataset, 32 subjects are involved in 40 trials of emotion-oriented music videos, where each music video lasts 60 s. DEAP uses the international 10–20 lead system to collect physiological data, which includes 32 channels of EEG signals and 8 channels of peripheral physiological signals. After each subject watched the music video, each subject was required to make self-assessments of valence, arousal, dominance, and liking on a scale of 1 to 9 by directly pressing the mouse at the corresponding location. In addition, the recorded data of each video of all subjects includes 3 s of baseline data and 60 s of experimental data. The data introduction of each subject is shown in Table 1. 32 channels of EEG signal data are used in our paper. According to the characteristics of the dataset and the representation of emotions in other studies, we use valence and arousal to represent emotions and define valence greater than 5 as positive, less than 5 as negative, arousal greater than 5 as high, and less than 5 as low.

### 4.2. Evaluation Metrics and Model Settings

The classification accuracy and F1-score are used to evaluate the CR-GCN method. Their calculation formulas are as follows

(19) $$\mathrm{accuracy}=\frac{TP+TN}{TP+TN+FP+FN}$$

(20) $$F1\text{-}\mathrm{score}=\frac{2\times TP}{2\times TP+FP+FN}$$

where TP, FP, TN, and FN are true positive, false positive, true negative, and false negative.

For hyperparameters of CR-GCN in all experiments, the Chebyshev polynomial order is *K* = 2, the last layer is softmax activation function, the remaining activation functions are ReLU activation function, the number of graph nodes is 32, the maximum number of iterations MAX is 1000, the dropout rate is set to 0.2, the batch size is 128, the learning rate $\eta =5\times 10^{-4}$, and the stop iteration threshold *e* = 0.0001. The model is trained and tested on NVIDIA GeForce GTX 1080 Ti and is implemented by Python 3.8.5 with PyTorch 1.7.0. For subject-dependent experiments, 80% of EEG data are used for training and 20% for testing for each subject. For subject-independent experiments, 80% of EEG data are used for training and 20% for testing for all subjects. Fivefold cross-validation with random strategy is adopted in all experiments.

### 4.3. Results

In this part, we conduct subject-dependent experiments, subject-independent experiments, and ablation experiments. In subject-dependent experiments, the training set and test set are from the same subject’s data. In subject-independent experiments, the training set and test set are not from the same subject’s data. In order to test the effectiveness and generalization of our method, we conducted two types of experiments instead of one.

#### 4.3.1. Subject-Dependent Experiments

In this part, the subject-dependent average classification results are shown in Table 2. The minimum, maximum, and average classification accuracies of the CR-GCN method on all subjects are 80.63%, 99.34%, and 94.69%, respectively, and the minimum, maximum, and average F1-score are 78.90%, 99.32%, and 94.40% on valence. The minimum, maximum, and average classification accuracies of the CR-GCN method on all subjects are 80.00%, 99.54%, and 93.95%, and the minimum, maximum, and average F1-score are 78.17%, 99.53%, and 92.78% on arousal.

Meanwhile, we compare CR-GCN with other methods in subject-dependent experiments. Results are shown in Table 3. CR-GCN is 3.89% higher than CNN and recurrent neural network (RNN) method [47] on valence and 2.92% higher on arousal. CR-GCN is 7.62% higher than the normalized frequency domain features (FREQNORM) and support vector machine (SVM) method [48] on valence and 6.97% higher on arousal. CR-GCN is 2.39% higher than multimodal residual long-short-term memory (MMResLSTM) [49] on valence and 1.08% higher on arousal. Furthermore, CR-GCN is 4.24% higher than ERDL [18] on valence and 3.35% higher on arousal.

According to the above results, we may conclude that CR-GCN is the most effective method. It also indicates that node feature normalization and combining adjacency matrices based on distance and functional connectivity are helpful for emotion recognition.

#### 4.3.2. Subject-Independent Experiments

In this part, the purpose of these experiments is to investigate whether CR-GCN can effectively reduce the differences among subjects so that we can obtain better emotion classification results in subject-independent experiments. Thus, we compare CR-GCN with other methods, and average classification accuracies are shown in Table 4. CR-GCN is 8.33% higher than correlated attention network (CAN) [50] on valence and 8.67% higher on arousal where the method applied a correlation attention network. CR-GCN is 13.68% higher than the Stack AutoEncoder (SAE) and LSTM method [51] on valence and 19.08% higher on arousal where the method used SAE and LSTM to realize emotion recognition. CR-GCN is 4.22% higher than emotion recognition based on hierarchy graph convolution network (ERHGCN) [35] on valence and 4.67% higher on arousal where the method extracted six types of features from five frequency bands and input them into HGCN model to realize emotion recognition. CR-GCN is 9.97% higher than ERDL [18] on valence and 8.19% higher on arousal where the method extracted differential entropy from EEG data to construct feature cubes and used GCN+LSTM to realize emotion recognition. CR-GCN is 10.95% higher than three-dimensional (3D) feature maps and CNN (3DCNER) [40] on valence and 8.93% higher on arousal where the method used 3D feature maps and CNN to realize emotion recognition. CR-GCN is 2.29% higher than spatial folding ensemble network (SFE-Net) [52] on valence and 1.52% higher on arousal where the method used an EEG-based symmetric spatial to realize emotion recognition.

ERDL [18] achieved good results in subject-dependent classification but not in subject-independent classification. Our proposed CR-GCN method achieves more than 93.46% in both classification effects, which shows the effectiveness and generalization of our method. It also shows that CR-GCN uses graph domain features of EEG data to obtain good classification results.

#### 4.3.3. Ablation Experiments

In this part, the purpose of these experiments is to explore the contribution of each important part in this method. The first ablation experiments are to explore whether node feature normalization can be beneficial to improve classification accuracy. The second ablation experiments are to explore whether our proposed adjacency matrix can help to improve classification accuracy. Fivefold cross-validation with random strategy is adopted in each experiment. In the following, CC represents the absolute value of correlation coefficient, the value 0.5 is randomly selected, and 0.98 is the result of our choice by repeated experiments.

(1) EEG Node Feature Normalization and No Normalization

**Figure 3:**
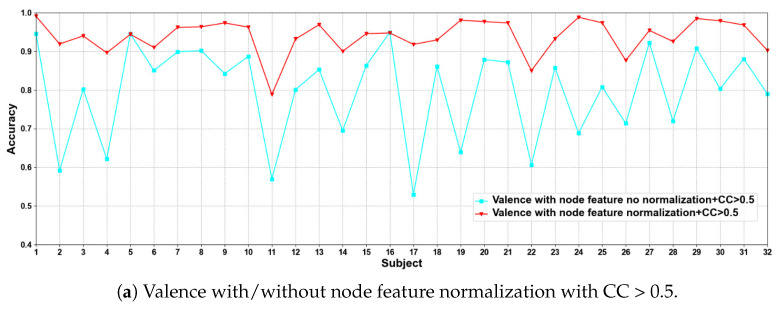

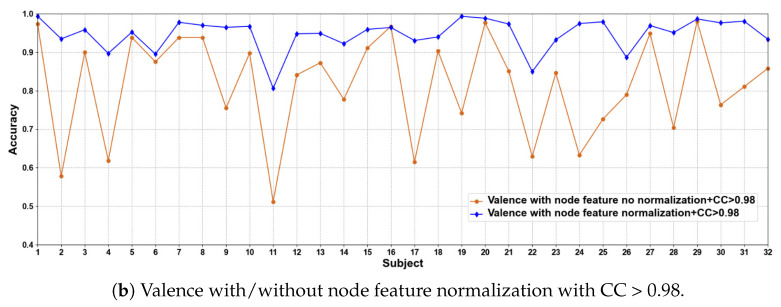
Classification accuracies on each subject on valence with/without normalization.

**Figure 4:**
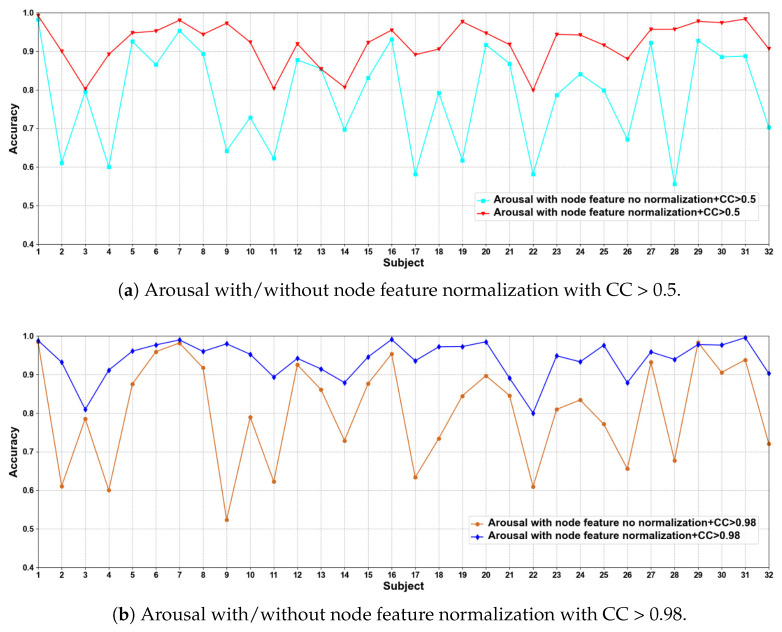
Classification accuracies on each subject on arousal with/without normalization.

In this part of the ablation experiments, we compare two methods to verify the results under the condition that the CC > 0.5 and 0.98, including node feature normalization and no normalization. The experimental results are shown in Figure 3 and Figure 4.

We compare the classification results of node feature normalization and no normalization. It can be seen that whether CC > 0.5 or CC > 0.98, the normalization of node feature is better than no normalization in most cases. In particular, the method has higher model performance accuracy when the CC > 0.98. At the same time, the average classification accuracies on all subjects are calculated. Results are shown in Table 5.

It can also be seen that the normalization of node feature is at least 13% higher than the method without normalization under the same conditions. We can also see that node feature normalization can improve classification accuracy. In particular, the method has higher classification accuracies when the CC > 0.98.

(2) The Different Construction Methods of Adjacency Matrix

**Figure 5:**
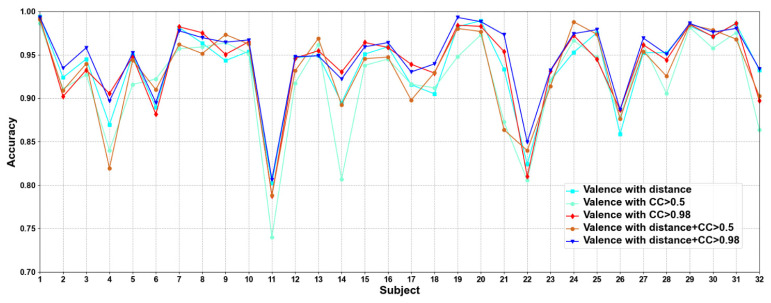
Classification accuracies on each subject on valence under construction of different adjacency matrices.

**Figure 6:**
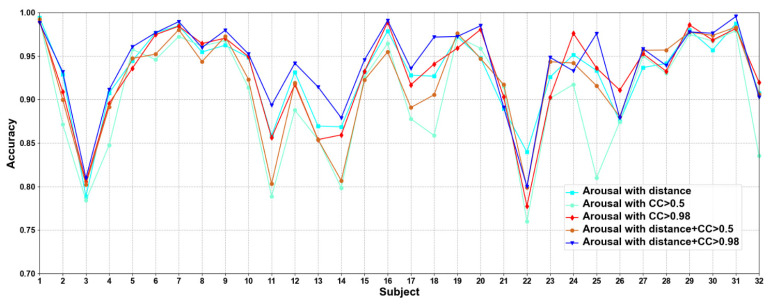
Classification accuracies on each subject on arousal under construction of different adjacency matrices.

In this part, we compare five methods to construct adjacency matrices, including distance-based method, the CC based on functional connectivity > 0.5 and 0.98, and the fusion of distance-based method and functional connectivity-based when the CC > 0.5 and 0.98. Results are shown in Figure 5 and Figure 6.

We compare different construction methods of adjacency matrix. It can be seen that although the effect of distance-based method is better than that functional connectivity-based method in most cases, there are also some subjects in the functional connectivity-based method achieve better performance than distance-based method, as in valence sub7, sub8, and sub10, etc., in arousal sub8, sub 9, and sub10, etc. This shows that only relying on the distance-based adjacency matrix (which tends to study local relationships) to construct the adjacency matrix without considering the correlation among channels (which tends to study global relationships) is not good for classification. It can be seen that the adjacency matrix based on the combination of distance and functional connectivity has the best classification effect when the CC > 0.98. At the same time, the average classification accuracies on all subjects are calculated. Results are shown in Table 6.

It can be seen that CC > 0.98 are higher than CC > 0.5 in functional connectivity-based methods, which indicates that too many added channel relationships are not conducive to model performance. The average classification accuracies of distance-based construction adjacency matrix are higher than that based on functional connectivity, which is the same as the research of Zhong et al. [17]. Since the adjacency matrix constructed by only distance-based method ignores the correlation among channels, we propose a method that combines distance and functional connectivity among channels to construct the adjacency matrix, in which it captures both local and global relationships of EEG channels. In addition, we can also see that the proposed adjacency matrix based on the fusion of distance and functional connectivity achieves better classification results, which again shows that the correct construction of adjacency matrix can effectively improve the performance of model.

## 5. Conclusions

A new method called CR-GCN by exploiting multiple relationships among EEG channels is proposed. Both subject-dependent and subject-independent experiments on DEAP are carried out, and the experimental results indicate that CR-GCN achieves better recognition performance than the state-of-the-art methods. In addition, ablation experiments show that the proposed normalization of node feature and adjacency matrix have significantly improved the performance of our method. The better emotion recognition result is attributed to the following points.

- The design of adjacency matrix captures local and global relationships among EEG channels and describes the relationships among EEG channels more accurately. The adjacency matrix design not only considers the biological topology but also considers the functional connectivity among EEG channels. Therefore, CR-GCN describes the relationships among EEG channels more accurately than ERDL [18];
- The graph representation of CR-GCN provides a better method to capture interchannel relationships and extract graph domain features, which is beneficial to realize emotion recognition.

Although the proposed CR-GCN has been shown to be a better method to deal with emotion recognition, the existing EEG emotion datasets are still relatively small in size and the data collection standards are not uniform (e.g., the selection of stimulus materials, the number of channels, and the time of data collection), which may restrict further performance improvements in our study. In the future, it is necessary to design data collection standards and build a larger EEG emotion database for emotion recognition.

## Figures and Tables

**Table 1:** The structure of DEAP.

Array Name	Array Shape	Array Contents
data	40 × 40 × 8064	video/trial × channel × data
labels	40 × 4	video/trial × label

**Table 2:** Subject-dependent average classification results.

Subject	Valence		Arousal	
	Accuracy (%)	F1-score (%)	Accuracy (%)	F1-score (%)
01	99.34	99.32	98.82	98.79
02	93.46	93.33	93.17	93.06
03	95.82	95.76	80.95	78.36
04	89.69	89.66	91.13	90.42
05	95.21	95.10	96.05	96.03
06	89.50	88.67	97.67	97.66
07	97.76	97.54	98.95	98.89
08	96.97	96.95	95.97	95.95
09	96.45	96.45	97.95	97.92
10	96.71	96.71	95.24	95.19
11	80.63	78.90	89.34	89.24
12	94.78	94.75	94.17	90.18
13	94.88	94.88	91.45	79.01
14	92.21	92.19	87.89	84.86
15	95.92	95.92	94.55	94.55
16	96.41	95.58	99.08	99.07
17	93.03	92.87	93.55	93.54
18	93.95	93.51	97.17	97.07
19	99.30	99.26	97.24	96.87
20	98.82	98.78	98.47	97.64
21	97.32	97.31	89.08	83.09
22	84.95	84.91	80.00	78.17
23	93.21	92.82	94.84	92.53
24	97.43	97.33	93.29	93.22
25	97.89	97.89	97.58	96.35
26	88.68	86.90	87.89	87.47
27	96.92	96.27	95.83	95.75
28	95.08	94.19	93.92	93.81
29	98.62	98.55	97.76	97.72
30	97.61	97.24	97.62	97.62
31	98.04	97.98	99.54	99.53
32	93.37	93.34	90.26	89.26
**Average**	**94.69**	**94.40**	**93.95**	**92.78**

**Table 3:** Average classification accuracies of other methods in subject-dependent experiments.

Methods	Valence (%)	Arousal (%)
CNN+RNN [47]	90.80	91.03
FREQNORM + SVM [48]	87.07	86.98
MMResLSTM [49]	92.30	92.87
ERDL [18]	90.45	90.60
CR-GCN	94.69	93.95

**Table 4:** Average classification accuracies of other methods in subject-independent experiments.

Methods	Valence (%)	Arousal (%)
CAN [50]	86.45	84.79
SAE+LSTM [51]	81.10	74.38
ERHGCN [35]	90.56	88.79
ERDL [18]	84.81	85.27
3DCNER [40]	83.83	84.53
SFE-Net [52]	92.49	91.94
CR-GCN	94.78	93.46

**Table 5:** Average classification accuracies on all subjects with/without node feature normalization.

Methods	Valence (%)	Arousal (%)
no normalization + CC > 0.5	79.66	78.58
normalization + CC > 0.5	93.09	91.99
no normalization + CC > 0.98	81.46	80.33
normalization + CC > 0.98	94.69	93.95

**Table 6:** Average classification accuracies on all subjects under construction of different adjacency matrices.

Methods	Valence (%)	Arousal (%)
distance	93.43	92.59
CC > 0.5	91.96	90.36
CC > 0.98	93.92	92.91
distance + CC > 0.5	93.09	91.99
distance + CC > 0.98	94.69	93.95

## Data Availability

The database used in this study is publicly available at websites: DEAP: http://www.eecs.qmul.ac.uk/mmv/datasets/deap/, accessed on 23 June 2022.
